# Toxicity associated with tuberculosis chemotherapy in the REMoxTB study

**DOI:** 10.1186/s12879-018-3230-6

**Published:** 2018-07-11

**Authors:** Conor D. Tweed, Angela M. Crook, Evans I. Amukoye, Rodney Dawson, Andreas H. Diacon, Madeline Hanekom, Timothy D. McHugh, Carl M. Mendel, Sarah K. Meredith, Michael E. Murphy, Saraswathi E. Murthy, Andrew J. Nunn, Patrick P. J. Phillips, Kasha P. Singh, Melvin Spigelman, Genevieve H. Wills, Stephen H. Gillespie

**Affiliations:** 1 MRC Clinical Trials Unit at University College London, London, UK; 2 Keya Medical Research Unit, Nairobi, Kenya; 3 University of Cape Town Lung Institute, Cape Town, South Africa; 4 TASK Applied Science, Cape Town, South Africa; 5 Division of Infection and Immunity, University College London, London, UK; 6 TB Alliance, New York, USA; 7 Division of Pulmonology, University of San Francisco, San Francisco, USA; 8 The Doherty Institute for Infection and Immunity, University of Melbourne and Royal Melbourne Hospital, Melbourne, Australia; 9 University of St Andrews Medical School, St Andrews, UK

**Keywords:** Tuberculosis, Toxicity, Clinical trials, Adverse events

## Abstract

**Background:**

The incidence and severity of tuberculosis chemotherapy toxicity is poorly characterised. We used data available from patients in the REMoxTB trial to provide an assessment of the risks associated with the standard regimen and two experimental regimens containing moxifloxacin.

**Methods:**

All grade 3 & 4 adverse events (AEs) and their relationship to treatment for patients who had taken at least one dose of therapy in the REMoxTB clinical trial were recorded. Univariable logistic regression was used to test the relationship of baseline characteristics to the incidence of grade 3 & 4 AEs and significant characteristics (*p* < 0.10) were incorporated into a multivariable model. The timing of AEs during therapy was analysed in standard therapy and the experimental arms. Logistic regression was used to investigate the relationship between AEs (total and related-only) and microbiological cure on treatment.

**Results:**

In the standard therapy arm 57 (8.9%) of 639 patients experienced ≥1 related AEs with 80 of the total 113 related events (70.8%) occurring in the intensive phase of treatment. Both four-month experimental arms (“isoniazid arm” with moxifloxacin substituted for ethambutol & “ethambutol arm” with moxifloxacin substituted for isoniazid) had a lower total of related grade 3 & 4 AEs than standard therapy (63 & 65 vs 113 AEs). Female gender (adjOR 1.97, 95% CI 0.91–1.83) and HIV-positive status (adjOR 3.33, 95% CI 1.55–7.14) were significantly associated with experiencing ≥1 related AE (*p* < 0.05) on standard therapy. The most common adverse events on standard therapy related to hepatobiliary, musculoskeletal and metabolic disorders. Patients who experienced ≥1 related AE were more likely to fail treatment or relapse (adjOR 3.11, 95% CI 1.59–6.10, *p* < 0.001).

**Conclusions:**

Most AEs considered related to standard therapy occurred in the intensive phase of treatment with female patients and HIV-positive patients demonstrating a significantly higher risk of AEs during treatment. Almost a tenth of standard therapy patients had a significant side effect, whereas both experimental arms recorded a lower incidence of toxicity. That patients with one or more AE are more likely to fail treatment suggests that treatment outcomes could be improved by identifying such patients through targeted monitoring.

## Background

Combination therapy for drug-susceptible tuberculosis (TB) using isoniazid (H), rifampicin (R), pyrazinamide (Z) and ethambutol (E) has been the standard of care for several decades. While there is recognised toxicity associated with some of the individual drugs (for example hepatotoxicity [1], peripheral neuropathy [2], and gastrointestinal upset [3]) there are few prospective studies of regimen-related toxicity [4–7]. Consequently, clinicians treating tuberculosis largely rely on retrospective or anecdotal evidence to guide their choices when patients experience adverse events.

Previous reports include female gender, alcohol use, human immunodeficiency virus (HIV) infection and certain ethnic groups [3–5, 8–11] as risk factors for experiencing adverse events during therapy. Accurate definition of the risk is confounded by the varying definitions of adverse events or drug-related toxicity, and uncertainty of the recording mechanism or its completeness. For example, ethambutol related impairment of visual acuity rates vary from 0.02% [12] and 9.4% [13] despite the importance of the complication and the simplicity of its measurement.

To overcome the difficulties of biased reporting we used the consistent adverse event reporting system used in the REMoxTB trial [14] to investigate drug related toxicity, as we believe that this is the most comprehensive source of safety data for standard tuberculosis therapy currently available. The aim of the paper was to accurately characterise the patients at greatest risk, the incidence and nature of the toxicity related to standard TB therapy, and to investigate the impact of toxicity on treatment outcomes. Additionally, the paper aimed to compare the incidence of toxicity for standard TB therapy and the two experimental arms in REMoxTB.

## Methods

### REMoxTB trial

The REMoxTB trial [14] was a double-blind, placebo-controlled, randomised phase III trial to investigate two experimental moxifloxacin (M)-containing treatment regimens to treat pulmonary tuberculosis. There were 1931 patients randomised between 2007 and 2012 with 655 assigned to the “isoniazid arm” (2MHRZ/2MHR), 636 assigned to the “ethambutol arm” (2EMRZ/2MR), and 639 allocated to standard TB therapy as a control (2EHRZ/4HR). Patients were followed for 18 months after randomisation. We included all randomised patients in the REMoxTB trial who had received at least one dose of their allocated treatment.

### Handling of safety data

Adverse events (AEs) were defined as any untoward medical occurrence in a patient administered the trial medication (with or without a causal relationship to the drugs) and were graded on a severity scale of 1 (least severe) to 4 (most severe) based on the Division of AIDS of the National Institute of Allergy and Infectious Diseases criteria [15]. The seriousness of an event (e.g. hospitalisation, life-threatening, death) irrespective of the severity was defined according to standard criteria. The local clinicians made the relatedness assessment for each event and those that were classified as “possibly”, “probably” or “definitely” related to drug therapy were considered to be “related” for the purposes of this analysis. Events that were assessed as either “unlikely related” or “not related” were considered to be “not related”. During the trial, sites were regularly monitored for data collection, and serious AEs (SAEs) were discussed in detail between the study medical monitor and the local clinician before being discussed in a safety board meeting with senior clinical specialists in the trial consortium to ensure quality control. Analyses and data handling were done using Stata statistical software version 14.1 (StataCorp, Texas).

### Baseline characteristics in standard therapy

Baseline characteristics for all the patients assigned to standard therapy, and the characteristics for those patients who experienced one or more related or unrelated grade 3/4 AE and those who experienced one or more related grade 3/4 AE were tabulated. Univariable logistic regression was performed using each of the baseline characteristics for patients receiving standard therapy in the table against a binary outcome for experiencing one or more total or related grade 3/4 AE. Those variables with a *p*-value of < 0.10 were manually selected for inclusion in a multivariable model, with age, gender and baseline weight included regardless of univariable *p* value due to their clinical relevance. Random-effects multivariable logistic regression was used to test for associations between the selected variables and grade 3/4 AEs with trial centre used as the panel variable to account for any effect from the individual sites.

### Adverse events in standard therapy over time

Incidence of grade 3/4 AEs and serious adverse events of any severity grade (SAEs) by treatment phase of standard therapy were categorised based on the grade 3/4 AE start date: intensive (weeks 0–8), continuation (weeks 9–26), and follow-up (week 27-month 18 after randomisation). MedDRA coding for System Organ Class and Preferred Term was used to identify the most common classes of adverse event. Patients were categorised based on the number of grade 3/4 AEs experienced in each phase of treatment. The mean number of SAEs per patient was calculated by dividing the total number of SAEs by the total number of patients with one or more SAE in each treatment phase. Patients who were withdrawn or died in the previous treatment phase were not included in later phases in order to present an accurate denominator for the number of patients at risk of an event in each of the three treatment phases. To illustrate the risk of grade 3/4 AE occurrence by time on standard treatment we constructed an Epanechnikov kernel smoothed hazard estimate with 95% confidence intervals and plotted the hazard function on the y-axis and the number of weeks from first dose on the x-axis.

### Adverse events and treatment outcomes on standard therapy

The number of grade 3/4 AEs reported by each patient taking standard therapy was related to their microbiological outcome at 18 months after starting treatment. Patients were scheduled for 8 weekly visits followed by 8 visits until 18 months after randomisation, and early morning and spot sputum samples were to be collected (where possible) at each visit. Sputum culture results were available for both solid and liquid media in the trial database.

For this analysis, cure was defined as patients who were culture negative at either 18 months or when they were last reviewed in the trial with at least two consecutive negative cultures on both solid and liquid media prior to their final negative result. This outcome was based exclusively on recorded culture status and was independent of the patient’s outcome in the original publication [14]. Patients were grouped according to whether they had experienced ≥1 total or related grade 3/4 AE, and the proportions of cured patients in these groups were tabulated. The Chi square test was used to test for significance and binary logistic regression with cure as an outcome was used to test the association between experiencing ≥1 total or related grade 3/4 AE and odds of cure. Sex, age, baseline weight and HIV status were included in a multivariable logistic model.

### Adverse events in all treatment arms

The incidence and classification of grade 3 or 4 adverse events (grade 3/4 AEs) and number of patients affected were calculated across the treatment arms according to the timing of the event: weeks 0–8 (EHRZ received on standard arm; MHRZ on isoniazid arm; EMRZ on ethambutol arm), weeks 9–17 (HR on standard arm; MHR on isoniazid arm; MR on ethambutol arm), weeks 18–26 (HR on standard arm; placebo on both isoniazid and ethambutol arms), and months 7–18 (no treatment administered for any arm and in trial follow-up). Grade 3/4 AEs were considered “clinically significant”. The proportion of patients with one or more grade 3/4 AE was compared across the treatment arms at each time window using the Chi square test. Time to first grade 3/4 AE in days after the first dose received was taken as the event of interest and Kaplan-Meier curves were constructed to illustrate the timing of grade 3/4 AEs in the three arms. The log rank test was used to compare the time to event in the standard therapy arm against the experimental arms individually.

### Ethics approval and participant consent

The REMoxTB study was carried out with approval from the ethics board at University College London, and this included approval for the use of data and samples collected in other studies to improve the diagnosis and treatment of tuberculosis. All randomised patients agreed to any data and samples collected as part of the trial being used in further studies to improve the diagnosis and treatment of tuberculosis, as stated on the informed consent form for the study. All the research activities and data collection for the study was compliant with the Helsinki Declaration and the principles of Good Clinical Practice.

## Results

### Baseline characteristics for patients allocated to standard therapy

Of 639 patients taking standard therapy 57 (8.9%) experienced one or more grade 3/4 AEs judged to be related to their treatment, compared to 45 (6.9%) of 655 in the isoniazid and 40 (6.3%) of 636 in the ethambutol arm (*p* = 0.21, see Tables 1 and 5). Baseline weight as a categorical variable (OR 0.79, 95% CI 0.65–0.97), female sex (OR 1.60, 95% CI 1.06–2.39), and HIV infection (OR 3.45, 95% CI 1.86–6.42) were significantly associated with ≥1 grade 3/4 AE in univariable logistic regression. However, only HIV infection was significantly associated with experiencing any grade 3 or 4 AE in a multivariable model (adjOR 3.43, 95% CI 1.82–6.49). Female sex (adjOR 1.97, 95% CI 0.91–1.83) and HIV infection (adjOR 3.33, 95% CI 1.55–7.14) were significantly associated with grade 3/4 AEs considered related to standard therapy after being selected for inclusion in the multivariable model (both *p* values < 0.05, see Table 2).

**Table 1:** Baseline characteristics of patients in the standard therapy arm

	Total	≥1 Grade 3/4 AEs	≥1 Related Grade 3/4 AEs
No of subjects	639	128	57
(% total)		(20.0%)	(8.9%)
Gender (%)			
Male	447	79 (17.7%)	31 (6.9%)
Female	192	49 (25.5%)	26 (13.5%)
Age			
<25yrs	187	37 (19.8%)	11 (5.9%)
25–35yrs	184	29 (15.8%)	20 (10.9%)
>35yrs	268	62 (23.1%)	26 (9.7%)
Baseline Weight			
<40kg	63	18 (28.6%)	8 (12.7%)
40–45kg	103	29 (28.2%)	14 (13.6%)
>45–55kg	254	40 (19.7%)	13 (5.1%)
>55–75kg	203	40 (19.7%)	22 (10.8%)
>75kg	16	1 (6.2%)	0 (0.0%)
Median	51.0	48.3	49.0
(IQR)	(45.0–57.7)	(42.3–57.0)	(42.5–58.0)
Ethnicity (%)			
Black	295	58 (19.7%)	26 (8.8%)
Asian	194	47 (24.2%)	20 (10.3%)
Mixed Race	140	21 (15.0%)	9 (6.4%)
Other	10	2 (20.0%)	2 (20.0%)
Smoking Hist (%)			
Never	298	63 (21.1%)	27 (9.1%)
Ex-smoker	155	29 (18.7%)	16 (10.3%)
Current	186	36 (19.3%)	57 (7.5%)
HIV Status			
Positive	46	20 (43.5%)	11 (23.9%)
Negative	593	108 (18.2%)	46 (7.8%)
Median CD4+ (IQR)	365.5 (307.0–456.0)	317.5 (267.5–458.5)	340.0 (267.0–488.0)
Cavities on CXR (%)	456	90 (19.7%)	32 (7.0%)
MGIT Median TTP (IQR)	114 (88–156)	111 (86–163)	118 (95–173)

The baseline characteristics for all patients in the treatment arm are listed along with the baseline characteristics for patients who experienced one or more grade 3 & 4 adverse event related or unrelated. Patients who experienced one or more grade 3 or 4 related adverse event are listed separately. Row percentages are included for each characteristic to show proportions of the total who experienced one or more adverse events

**Table 2:** Logistic Regression output to test the association between baseline characteristics and the risk of experiencing one or more grade 3 or 4 Adverse Event (AE) on standard TB therapy

	Univariable analysis			Multivariable analysis		
Baseline characteristic	OR	95% CI	*P* value	adjOR	95% CI	*P* value
Female sex	1.60	1.06–2.39	0.02	1.36	0.87–2.13	0.18
	(2.10)	(1.21–3.65)	(0.01)	(1.97)	(0.91–1.83)	(0.03)
Age	1.13	0.90–1.43	0.30	1.15	0.90–1.47	0.25
	(1.25)	(0.89–1.74)	(0.20)	(1.29)	(0.91–1.83)	(0.16)
Baseline weight	0.79	0.65–0.97	0.02	0.80	0.63–1.03	0.08
	(0.86)	(0.65–1.14)	(0.30)	(0.90)	(0.67–1.21)	(0.49)
Ethnicity						
Black	Reference	***	***	***	***	***
Asian	1.31	0.84–2.02	0.23	***	***	***
	(1.19)	(0.64–2.20)	(0.58)			
Mix race	0.72	0.42–1.24	0.24	***	***	***
	(0.71)	(0.32–1.56)	(0.40)			
Smoking Hist.						
Never	Reference	***	***	***	***	***
Ex-smoker	0.86	0.53–1.40	0.54	***	***	***
	(1.16)	(0.60–2.22)	(0.66)			
Current	0.90	0.57–1.42	0.64	***	***	***
	(0.82)	(0.42–1.60)	(0.56)			
HIV positive	3.45	1.86–6.42	< 0.001	3.43	1.82–6.49	< 0.01
	(3.74)	(1.78–7.84)	(< 0.001)	(3.33)	(1.55–7.14)	(< 0.01)
Cavities on CXR	1.03	0.62–1.71	0.92	***	***	***
	(0.62)	(0.31–1.21)	(0.16)			
Baseline TTP	1.24	0.55–2.82	0.60	***	***	***
	(1.39)	(0.47–4.09)	(0.55)			

Univariable odds ratio (OR) shown and characteristics with *p* value < 0.10 were manually added to a multivariable model to test for association. A random-effects multivariable logistic regression model was used with trial centre as the panels. ORs for experiencing any grade 3 or 4 AE shown with ORs for experiencing one or more related grade 3 or 4 AE provided in brackets. Baseline variables indicated in the table where necessary. Age and weight were entered as categorical variables shown in Table 2, and baseline time to MGIT positive (TTP) was entered as binary variable of below or above/equal to the median. Age, gender and baseline weight were included in the multivariable model regardless of *p* value due to clinical significance

***No analysis performed

### Adverse events in standard therapy

**Fig. 1:**
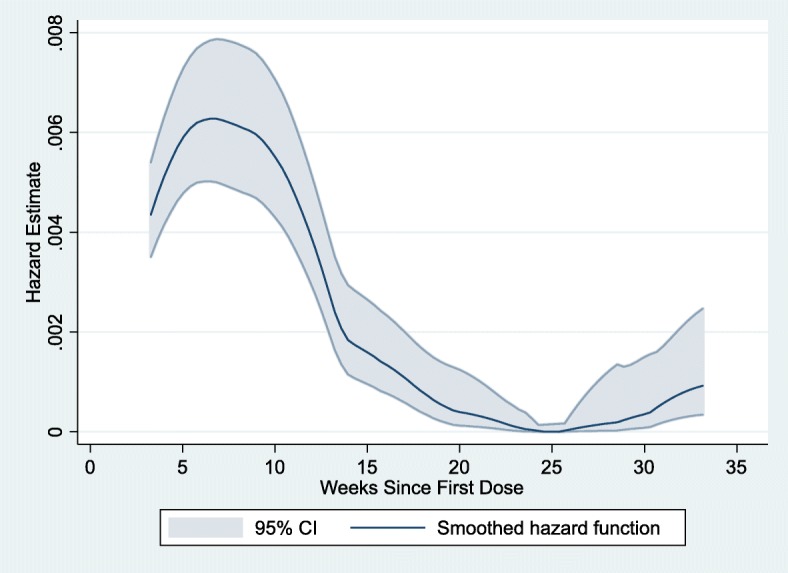
Hazard Curve for Related Grade 3 & 4 Adverse Events. Hazard Curve for Grade 3 or 4 Related Adverse Events According to Number of Weeks Since First Dose of Standard Therapy. Hazard function for the occurrence of a grade 3 or 4 related adverse event (with 95% confidence intervals) is plotted on the y axis, with the number of weeks following the first dose of standard tuberculosis therapy on the x axis. The rise in the hazard function after week 25 is accounted for by 2 events reported as “possibly” related to study drug

Among the 113 related grade 3/4 AEs in the standard therapy group 80 (70.8%) were reported in the intensive phase of treatment (month 1 &2) as shown in Table 3 and illustrated in Fig. 1. Of the 57 patients who experienced ≥1 related grade 3/4 AE on treatment, 47 (82.5%) experienced an event in the intensive phase. The related adverse events most commonly reported were elevated liver enzymes (38 of 38 “hepatobiliary” events), arthralgia (15 of 22 “musculoskeletal” events), and diabetic complications (4 of 12 events attributed to “metabolism & nutrition”) (see Table 3). There was one case of deterioration in visual acuity reported in the standard arm (data not shown).

**Table 3:** Events in standard arm by treatment phase

	Intensive phase (Month 0–2) *n* = 639	Continuation phase (Month 3–6) *n* = 596	Follow Up phase (Month 7–18) *n* = 569
Total Grade 3 & 4 AEs Reported	135	62	53
Related	80	29	4
(% Total)	(59.3%)	(46.8%)	(7.5%)
No of Grade 3 AEs Reported	100	48	33
Related	50	23	3
(%Grade3)	(50.0%)	(57.5%)	(9.1%)
No. Grade 4 AEs Reported	35	14	20
Related	30	6	1
(%Grade4)	(85.7%)	(42.9%)	(5.0%)
System Organ Class of Related Events*			
Hepatobiliary	25	13	0
Musculoskeletal	15	7	0
Metabolism & Nutrition	9	1	2
Blood & Lymphatic	5	2	1
No of Related Grade 3 or 4 AEs per Patient			
0	592	581	566
1	33	12	2
2	10	1	1
≥ 3	4	2	0
No of Patients with ≥ 1 SAE (% n)	32 (5.0%)	18 (3.0%)	20 (3.5%)
No of Patients with ≥ 1 Related SAE (%n)	17 (2.7%)	3 (0.5%)	2 (0.4%)
Mean No of SAEs per Patient	1.78	1.39	1.60
No of Withdrawals	38	26	1
No of Deaths	5	1	10

The number of grade 3 & 4 adverse events (total and related only) recorded in each treatment arm are shown with percentage of the total number of similar events across all treatment phases. Most common System Organ Classes for grade 3 & 4 adverse events are tabulated by treatment phase, along with tallies of patients in each phase split by the number of grade 3 or 4 adverse events experienced in the treatment phase. Serious adverse events in each phase are also shown, regardless of their severity grading. The denominator for each treatment phase was determined by subtracting the number of withdrawals and deaths from the denominator in the previous phase. The treatment phases were not independent and the same patient could appear in all three of the phases. *Note that some events excluded because of undocumented onset date

While the majority of SAEs were reported during therapy 10 of the 16 deaths in this treatment arm occurred after treatment was completed (see Table 3). The most common causes of death were trauma, suicide or unknown cause but presumed to be violent (8 of 16), and related to TB disease (3 of 16). Right heart failure, sepsis of unknown origin, and uncontrolled hypertension accounted for the remaining three deaths. None of the deaths in the standard therapy group were assessed as related to treatment.

### Treatment outcomes for patients on standard therapy

Patients who had one or more total or related grade 3/4 AE were less likely to achieve microbiological cure compared to patients who did not experience a grade 3/4 AE (see Table 4). Of patients taking standard TB therapy, 21.1% (27 of 128) of patients with ≥1 grade 3/4 AE were not cured, compared to 9.2% (47 of 511) of patients who did not experience any grade 3/4 AE. Similarly, 26.3% (15 of 57) of patients who experienced ≥1 grade 3/4 AE considered related to treatment did not achieve cure compared to 10.1% (59 of 582) of patients who did not experience a related grade 3/4 AE (*p* value < 0.001).

**Table 4:** Rates of microbiological cure according to number of Grade 3 or 4 adverse events experienced by patients taking standard TB therapy

	No. of grade 3/4 AEs	Microbiological cure	No microbiological cure	Total
Total	0	464 (90.8%)	47 (9.2%)	511
	≥1	101 (78.9%)	27 (21.1%)	128
Related only	0	523 (89.9%)	59 (10.1%)	582
	≥1	42 (73.7%)	15 (26.3%)	57

Patients are grouped by the number of AEs they experienced in the trial. The number of patients who were either cured or not cured of their TB (based on definition given in Methods section) are displayed with row percentages (Chi square test *p* value < 0.001)

Experiencing ≥1 related or unrelated grade 3/4 AE was significantly associated with not being cured of TB in a multivariable logistic regression model (adjOR 2.60, 95% CI 1.52–4.46, *p* < 0.001). A similar relationship was seen between ≥1 related-only grade 3/4 AE and an outcome of not cured (adjOR 3.11, 95% CI 1.59–6.10, *p* value < 0.001). The multivariable model included sex, age and baseline weight (clinical significance) and HIV status (due to earlier reported association with AE incidence).

### Adverse events across all treatment arms over time

Most grade 3/4 AEs occurred during the intensive phase for all regimens (see Table 5) with 80 (73.4%), 51 (81.0%) and 44 (67.7%) related grade 3/4 AEs during the intensive phase in the standard, isoniazid, and ethambutol arms respectively. Both experimental arms had lower numbers of related grade 3/4 AEs (64 and 66 in the isoniazid and ethambutol arms vs 113 during standard therapy). There was a significant difference in the proportion of patients experiencing ≥1 related grade 3/4 AE in the intensive phase (p value 0.03) with the smallest proportion in the ethambutol arm (25 of 636 [3.9%], see Table 5). In all treatment arms the most common type of related grade 3/4 AEs were “hepatobiliary disorders” (40.7% in standard therapy, and 42.2% & 37.9% in isoniazid & ethambutol arms).

**Table 5:** Comparing adverse events in treatment arms

		Standard Arm (2EHRZ/4HR) *n* = 639	Isoniazid Arm (2MHRZ/2MHR) *n* = 655	Ethambutol Arm (2EMRZ/2MR) *n* = 636	*P* value
Intensive Phase (Weeks 0–8)	Patients with ≥ 1 Grade 3/4 AEs (Tot No AEs)	85 (135)	83 (119)	66 (114)	0.24
	Patients with ≥ 1 Related Grade 3/4 AEs (Tot No AEs)	47 (80)	36 (51)	25 (44)	0.03
Continuation Phase (Weeks 9–17)	Patients with ≥ 1 Grade 3/4 AEs (Tot No AEs)	29 (47)	25 (32)	26 (37)	0.81
	Patients with ≥ 1 Related Grade 3/4 AEs (Tot No AEs)	14 (27)	9 (9)	16 (19)	0.32
Continuation/Placebo Phase (Weeks 18–26)	Patients with ≥ 1 Grade 3/4 AEs (Tot No AEs)	12 (15)	13 (17)	17 (21)	0.57
	Patients with ≥ 1 Related Grade 3/4 AEs (Tot No AEs)	2 (2)	3 (3)	2 (2)	0.88
Total*	Patients with ≥ 1 Grade 3/4 AEs (≥1 Related Grade 3/4 AEs)	128 (57)	103 (45)	94 (40)	0.40 (0.21)
	Total Grade 3/4 AEs (Related Grade 3/4 AEs only)	250 (113)	217 (64)	209 (66)	

The number of patients experiencing one or more grade 3 or 4 adverse event, and those who experienced events considered related to treatment only, are shown according to the treatment phase and study arm in the trial. The numbers of events are shown in brackets. The Chi square test was used to test for significant differences between the treatment arms for the proportions of patients who experienced ≥1 event in each treatment phase, for both total and related-only grade 3 or 4 AEs. Number of patients shown is number for that treatment window: 4 patients with ≥1 related AE appear in more than one time window on standard therapy, and 3 patients in both the isoniazid and ethambutol arms. Additionally, two patients excluded from the total count on the standard arm as no start date for AEs recorded *AEs that occurred in the follow-up phase (months 7–18) included in total

**Fig. 2:**
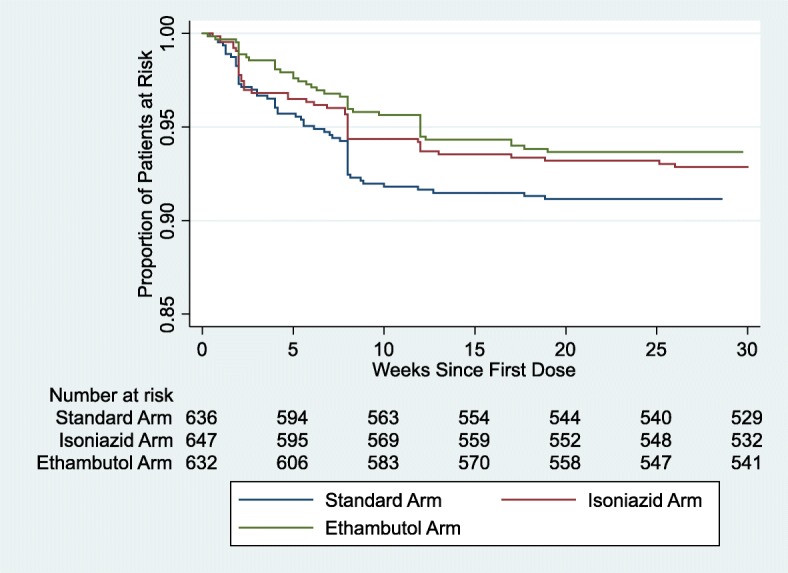
Related Grade 3 or 4 Adverse Events By Treatment Arm. Kaplan Meier Curves for Time to First Event for Related Grade 3 or 4 Adverse Events in the Treatment Arms. The time to first event is plotted for all the patients at risk in the standard (blue), isoniazid (red) and ethambutol (green) arms. The y axis plots the proportion of the patients still at risk, and the risk table presents this numerically. The data was censored at 200 days after the first dose for all three arms, and there was no significant difference between the isoniazid arm (*p* = 0.19) or the ethambutol arm (*p* = 0.07) when compared to the standard therapy using the log rank test

There was no difference in either the overall total or related total of grade 3/4 AEs between the three treatment arms during weeks 18–26 when patients in the experimental regimens were receiving placebo. The Kaplan Meier curves in Fig. 2 illustrate the majority of events occurring in the intensive phase followed by a plateau from approximately 9 weeks after starting treatment (log rank *p* = 0.19 for comparing standard therapy and isoniazid arm; *p* = 0.07 for standard therapy and ethambutol arm). The drop seen at 8 weeks of treatment in the number of patients at risk was driven by one site reporting 40 grade 3/4 AEs (30 considered related) from all treatment arms in a 30-week time window of the trial between May and December 2010 (the site reported a total of 146 grade 3/4 AEs). 36 of 40 (90%) of these events were reported in the intensive phase of the patient’s treatment.

## Discussion

In a study encompassing a large number of drug-sensitive TB patients from across the world there is evidence that almost a tenth of patients experienced serious side effects due to their TB medication. The existing literature quotes a rate of approximately 5–20% for significant toxicity from standard TB therapy [5, 9, 16–21] and hepatotoxicity is the most frequently detected [1, 22, 23]. The liver enzyme profile on treatment for standard TB therapy and the experimental arms in REMoxTB has been described in more detail elsewhere [24]. As the exclusion criteria removed those with severe disease or concomitant diseases, our estimate must be considered a minimum [14], however the follow-up period was comparable to two other recent large tuberculosis trials [25, 26].

We observed that most of the AEs in standard therapy occurred in the intensive phase. The explanation for this is uncertain, and could involve a degree of survivorship bias or increased tolerance of side effects, but may relate to the presence of pyrazinamide in all three regimens. Pyrazinamide is a drug with a well-recognised toxicity profile [11], while ethambutol (the other drug only present for the intensive phase) has few reported side effects [27]. Additionally, hepatotoxicity and arthralgia were among the most common events and these are frequently reported side effects of pyrazinamide [28, 29]. The sterilising activity of pyrazinamide makes it an essential component of standard therapy [30], but there is still some uncertainty surrounding its ideal dosing [31] and there is evidence of a dose-response relationship with toxicity [32]. There is a pressing need to direct more research to optimise the most effective and least toxic dose alongside the other components of the standard regimen [33].

It is perhaps significant there was little difference in the number of related AEs in months 5 and 6 between those receiving active treatment and those on placebo. This could emphasise the importance of TB induced pathology on the presence and reporting of significant medical events. Reducing toxicity associated with medication is one of the factors driving the development of shorter treatment regimens for TB [34], however this finding suggests that concerns about toxicity may not be as important as previously thought. While the experimental arms were less toxic, it should be noted that they were also less effective. It was notable that both experimental regimens were less toxic and both of these reduced the bacterial load more quickly than standard regimen [14]. Whether there is a causal relationship between these observations is not known. This means, perhaps, that the motivation for shortening treatment needs to focus around patient acceptability and logistical benefits of few doses, visits to clinics and enhanced adherence.

We found female patients and HIV-positive patients to be at significantly higher risk of toxicity. Existing guidelines acknowledge the issues surrounding TB-HIV co-infection [35, 36], but these do not reference female gender as a risk factor for a more complicated treatment course (outside of pregnancy). It is unclear if reporting bias has played a role in AE recording for the trial, as there have been discrepancies noted between the genders in regards to healthcare-seeking behaviour previously [37, 38]. Nonetheless, clinicians should consider closer monitoring of both HIV-positive and female patients taking HRZE, especially in the intensive phase of treatment.

Those patients reporting one or more related grade 3/4 AE were more likely to fail to achieve sustained sputum culture negative status. This is an important observation and emphasises the need to detect toxicity early and manage it properly. The reasons for this difference in outcome is uncertain and would merit further investigation in prospective studies. It may be that better management of drug toxicity in tuberculosis treatment could deliver better outcomes.

It is notable that the majority of deaths occurred after completing treatment and were unrelated to trial medication, emphasising the importance of social context of TB infection. It may be that this is due to other underlying conditions that also contribute to a poor outcome or that experiencing toxicity reduces adherence to therapy. This relationship may explain why the cure rate with standard therapy for drug-sensitive disease can be as low as 80% in real-world settings [39].

This study is limited by innate reporting bias and reliance on a subjective assessment of severity in many cases (for example, pain scores). An example of this is the reporting activity at one site in the trial. After a trial pause this site reported almost one third of its total grade 3/4 AEs, and assessed 75% of them as being related to treatment in a 30 week period. Attributing causality to AEs has been shown to produce unreliable and subjective data [40] and caution has been advised when using trial data to evaluate drug safety profiles [41]. Given the proximity of the recent pause in trial recruitment it could be that there was concern over the safety of the experimental regimens and that in a double-blind trial this translated into a lower threshold to both report events and to attribute causality to the drugs.

While there is still merit in using AEs to investigate drug safety profiles, the often subjective nature of the reporting is a limitation. We are also aware of the potential dangers of drawing conclusions based on relatedness assessments for AEs [40, 42] and to this end have presented both total and related AEs in the analysis. Overall, the careful and consistent way in which data were recorded for this large number of patients does mean, however, that we are able to generate important observations and suggest future research.

In this paper we have shown that most adverse events occur in the intensive phase of treatment with female patients and those who are HIV positive constituting a demographic that should be closely monitored for toxicity. We have also found that those who experience clinically significant drug related-toxicity while taking standard TB therapy are at greater risk of failing treatment. From this we conclude that we need to improve our methods of detecting and managing patients experiencing toxicity, and that there is real need for novel drugs with more favourable toxicity profiles. Our data provide an evidence base to plan future research and to support improved treatment guidelines. Tuberculosis remains a global health threat, predominantly affecting a vulnerable and disadvantaged population, and this paper illustrates the need for clinicians to be quick to respond to side effects from treatment to ensure their patients have the best chance of achieving a cure.
